# Supplementary material for: Modeling Self-Rollable Elastomeric Films for Building Bioinspired Hierarchical 3D Structures
Source: Int J Mol Sci. 2022 Jul 30;23(15):8467. doi: 10.3390/ijms23158467 (PMC9369037; doi:10.3390/ijms23158467)
Supplement: Supplementary file 1 [file ijms-23-08467-s001.zip › ijms-1755782-supplementary.pdf]

# Supporting Information

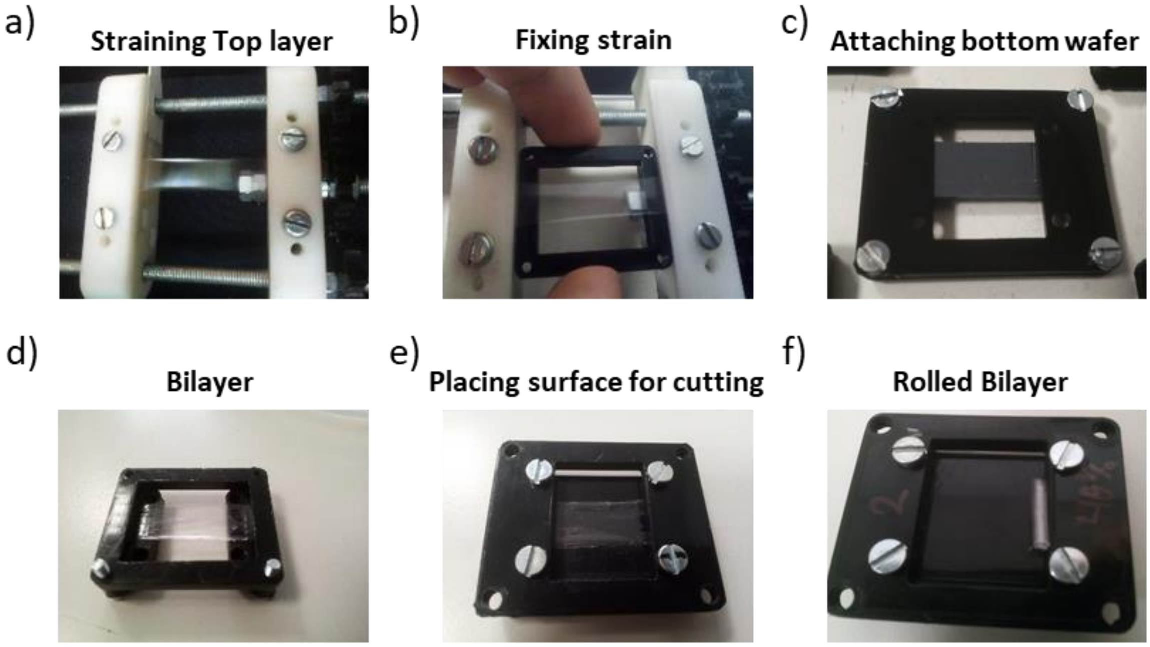


*Figure S1: Scheme of the fabrication process of rolled bilayers. a) the top layer is stretched for the desired amount (from 20 % to 60 %, in this work), then b) the top layer is blocked at a fixed strain. c) Application of the bottom layer to the top one to achieve the bilayer shown in d). e) A support is placed below the bilayer to enable the cutting on one side of the bilayered membrane, which rolls f) without external constraints.*

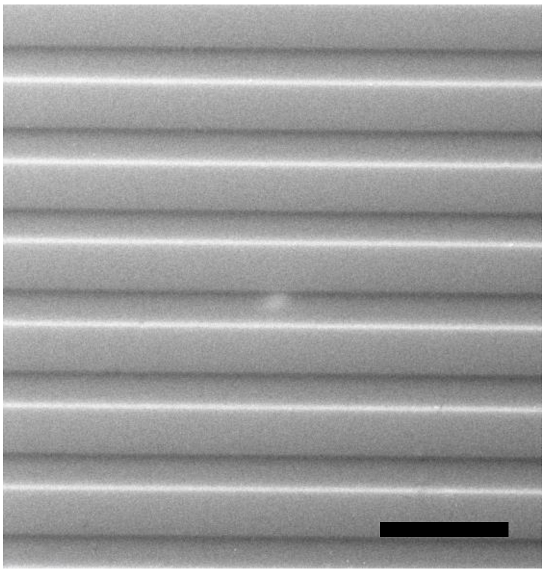

*Figure S2: SEM image of the topographic features built on the top layer. Scale bar is 30 µm.*

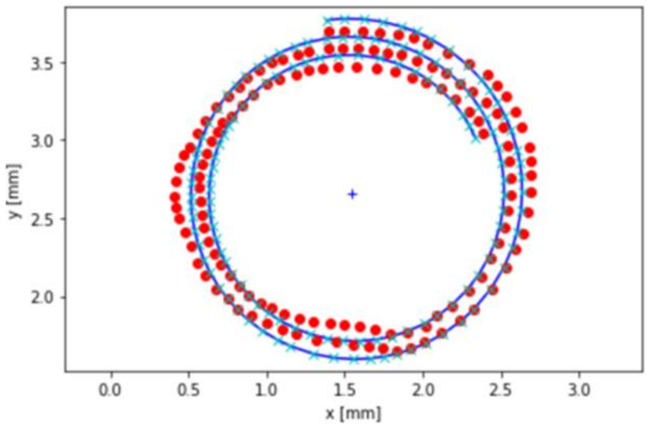


*Figure S3: Example of a spiral fitting process applied to an image of a rolled structure acquired with an optical microscope. Spiral fit process: in red, points along the midline of an experimental sample are shown; in blue, the fit with an Archimedean spiral fitting process was performed to extract the inner and outer radii of the samples. The spiral fitting procedure started with the acquisition of a microscope image of the samples. These images were later processed in MATLAB, where a set of points along the midline of the bilayers were collected. An algorithm compared the set of points with many Archimedean spirals and found the one that best fit the experimental data. In the end, we extracted a set of inner and outer diameters from the spirals that best fit the data.*

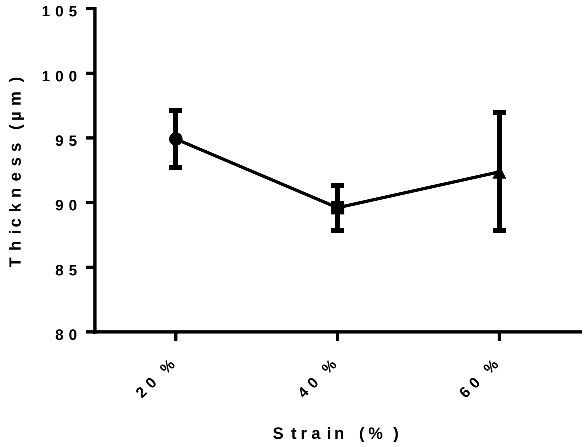


*Figure S4: Thickness vs. strain applied (20 %, 40 % and 60 %). The presence of the pillars (height: 23.6 ± 0.4 µm) has not been considered in this graph. Differences between the experimental groups were not statistically significant.*

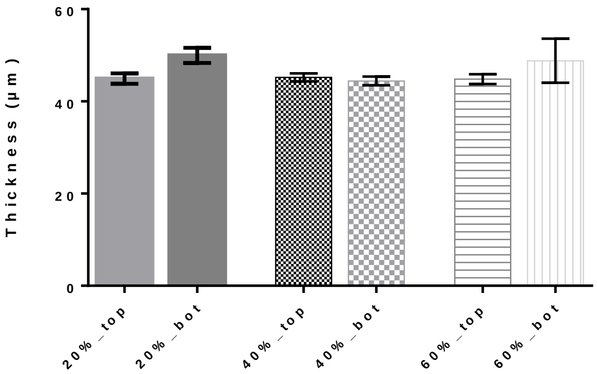


*Figure S5: Thickness of the top (_top) and bottom (_bot) layers for the different applied strain values. The presence of pillars (23.6 ± 0.4 µm) has not been considered in this graph. Differences between the experimental groups were not statistically significant.*

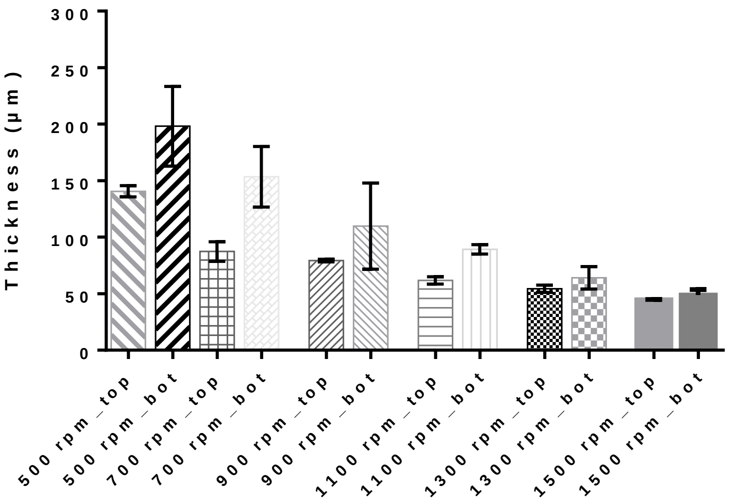


*Figure S6: Thickness of the top (_top) and bottom (_bot) layers for different spin speed values, applying a strain mismatch of 60% at the top layer. The presence of pillars (23.6 ± 0.4 µm) has not been considered in this graph.*

*Table S1: Comparison of the inner and outer radii of model predictions and experimental data for the inner unit's geometry.*

| **Mean value outer spiral** | **Experiment (mm)** | **Model (mm)** | **Error (%)** |
|---|---|---|---|
| Inner radii | 1.314 | 1.44 | 9.6 |
| Outer radii | 1.393 | 1.535 | 10.19 |

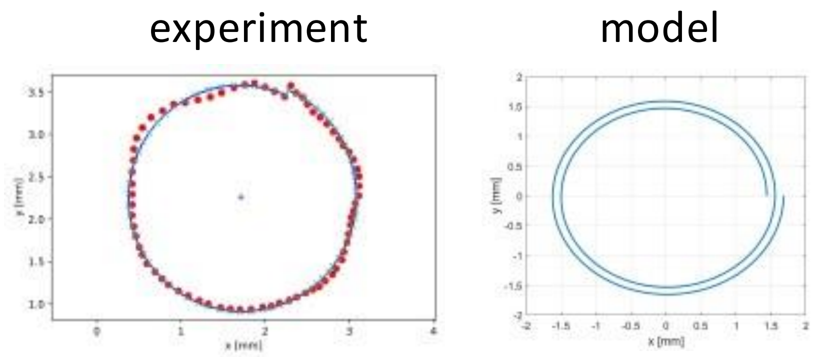


*Figure S7: Spiral fitting characterization for the outer roll of the hierarchical spiral.*

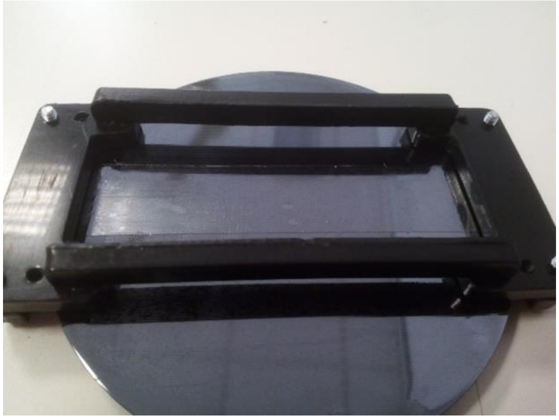

*Figure S8: Image of the custom-made stretching setup adapted for the fabrication of the hierarchical structure.*

## Spontaneous curvature of the bilayer

Let $\mathbf{e}_i$, $i = 1, 2, 3$ be an orthonormal basis corresponding to the Cartesian coordinates $x_i$. We consider two separate layers with (undeformed) lengths $l_r$ (top) and $l_0<l_r$ (bottom) and thicknesses $(1-\beta)h_0$ and $\beta h_0$, $0<\beta<1$, respectively. The bottom layer is then stretched along the direction $\mathbf{e}_1$ by $\lambda=l_r/l_0=1+\varepsilon$, with $\varepsilon = 0.2, 0.4, 0.6$ the pre-strain, and glued to the top layer. Hence, the bilayer attains its reference configuration, where $x_1 \in [-\, l_r/2, l_r/2]$ and $x_3 \in [-\, h_r/2, h_r/2]$ span the longitudinal and the thickness directions, respectively. This deformation process may be described by the deformation gradient for the bottom layer:

$$\boldsymbol{F}_0^b = \lambda \boldsymbol{e}_1 \otimes \boldsymbol{e}_1 + \frac{1}{\sqrt{\lambda}}(\boldsymbol{e}_2 \otimes \boldsymbol{e}_2 + \boldsymbol{e}_3 \otimes \boldsymbol{e}_3), \qquad \text{(S1)}$$

where we have assumed incompressibility, while the deformation gradient for the top layer is $\boldsymbol{F}_o^t = \boldsymbol{I}$. Following [21], we then represent the plane strain bending motion from the reference configuration through the deformation gradient:

$$\boldsymbol{F} = \lambda_r(r)\boldsymbol{e}_r \otimes \boldsymbol{e}_3 + \lambda_\theta(r)\boldsymbol{e}_\theta \otimes \boldsymbol{e}_1 + \boldsymbol{e}_2 \otimes \boldsymbol{e}_2, \quad \lambda_r(r) = \frac{l_r}{2\bar{\theta}r}, \quad \lambda_\theta(r) = \frac{l_r}{\lambda_r(r)} \qquad \text{(S2)}$$

where $2\bar{\theta}$ is the bending angle, $\mathbf{e}_r$ -$\mathbf{e}_\theta$ is a polar basis in the bending plane $\mathbf{e}_1$ -$\mathbf{e}_3$. Here, we have assumed the bending to be isochoric and $r$ is the radial coordinate, with $r_i^t, r_i^b (r_e^t, r_e^b)$ the coordinates of the intradoses (extradoses) of the top and bottom layers, respectively. The total deformation gradient that causes stress within the bilayer is thus $\mathbf{F}\mathbf{F}_0^{\mathrm{b}}$ for the bottom layer and $\mathbf{F}$ for the top layer. Considering a Neo-Hookean constitutive response for both layers, the Cauchy stress in each layer reads:

$$\mathbf{T}^t = G_t(\lambda_r^2 \boldsymbol{e}_r \otimes \boldsymbol{e}_r + \boldsymbol{e}_\theta \otimes \boldsymbol{e}_\theta) - p_t\mathbf{I} + G_b\boldsymbol{e}_2 \otimes \boldsymbol{e}_2, \qquad \text{(S3)}$$

$$\mathbf{T}^b = G_b[\frac{\lambda_r^2}{\lambda}\boldsymbol{e}_r \otimes \boldsymbol{e}_r + (\lambda_\theta\lambda)^2\boldsymbol{e}_\theta \otimes \boldsymbol{e}_\theta] - p_b\mathbf{I} + \frac{G_b}{\lambda}\boldsymbol{e}_2 \otimes \boldsymbol{e}_2, \qquad \text{(S4)}$$

Where $p_t$, $p_b$ are the Lagrange multipliers associated to the incompressibility constraints:

$$(1-\beta)h_0 l_r = \bar{\theta}((r_e^t)^2 - (r_i^t)^2), \qquad \frac{1}{\sqrt{\lambda}}\beta h_0 l_r = \bar{\theta}((r_e^b)^2 - (r_i^b)^2), \tag{S5}$$

with $r_e^b = r_i^t$, and $G_t$, $G_b$ are the shear moduli of the top and the bottom layer, respectively. At this point, it is convenient to introduce the change of variables

$$\omega = 4\bar{\theta}\frac{h_0}{l_r}, \qquad \gamma_i^t = \frac{\bar{\theta}(r_i^t)^2}{h_0 l_r}, \qquad z = \frac{l_r}{2\bar{\theta}} = \frac{2\mathrm{h}_0}{\omega}, \tag{S6}$$

With this, the incompressibility constraints eq. (5) may be manipulated to provide the following relations:

$$r_e^t = z\sqrt{\omega(\gamma_i^t + 1 - \beta)}, \qquad r_i^b = z\sqrt{\omega(\gamma_i^t - \beta/\sqrt{\lambda})}, \tag{S7}$$

Upon introducing the radial $T_r^{t,b} = \mathbf{T}^{t,b}\boldsymbol{e}_r \cdot \boldsymbol{e}_r$ and hoop $T_\theta^{t,b} = \mathbf{T}^{t,b}\boldsymbol{e}_\theta \cdot \boldsymbol{e}_\theta$ stresses and upon solving the balance of forces:

$$\frac{\partial T_r^{t,b}}{\partial r} + \frac{T_r^{t,b} - T_\theta^{t,b}}{r} = 0, \qquad \frac{\partial T_\theta^{t,b}}{\partial \theta} = 0, \tag{S8}$$

for each layer, along with the boundary/interface conditions:

$$T_r^t(r_e^t) = 0, \qquad T_r^b(r_i^b) = 0, \qquad T_r^t(r_i^t) = T_r^b(r_e^b) \tag{S9}$$

the constitutive equations eq. (4) and the incompressibility conditions eq. (5), we obtain the pressure terms and the following relation from the interface condition:

$$\omega^2 = \frac{1}{\alpha(1-\beta)+\beta\lambda^{3/2}}\left[\frac{\alpha}{\gamma_i^t} - \frac{\alpha}{\gamma_i^t+1-\beta} + \frac{1}{\lambda}\left(\frac{1}{\gamma_i^t-\beta/\sqrt{\lambda}} - \frac{1}{\gamma_i^t}\right)\right], \tag{S10}$$

with $\alpha = G_t/G_b$. Finally, by imposing that the resultant bending moment vanishes, i.e.:

$$M = \int_{r_i^b}^{r_e^b} rT_\theta^b dr + \int_{r_i^t}^{r_e^t} rT_\theta^t dr = 0, \tag{S11}$$

and accounting for eq. (10), we get a non-linear equation in the unknown $\gamma_i^t$:

$$2\alpha\log\frac{1-\beta+\gamma_i^t}{\gamma_i^t} + \frac{2}{\lambda}\log\frac{\gamma_i^t}{\gamma_i^t - \beta/\sqrt{\lambda}} +$$

$$-\frac{(\beta(1-\beta+\gamma_i^t)+\alpha(-1+\beta)\beta\lambda-\alpha(-1+\beta)\gamma_i^t\lambda^{3/2})(\alpha(-1+\beta)(-1+\beta-2\gamma_i^t)-\beta^2\lambda+2\beta\gamma\lambda^{3/2})}{\lambda(-1+\beta-\gamma_i^t)\gamma_i^t(\beta-\gamma_i^t\sqrt{\lambda})(\alpha-\alpha\beta+\beta\lambda^{3/2})}=0$$

(S12)

Once solved (numerically) for $\gamma_i^t$, the latter equation allows to compute $\omega$ from eq. (10). Then, using eqs. (6)-(7), we may finally estimate the spontaneous curvature of the bilayer as:

$$k_s=\frac{2}{r_i^b+r_e^t}, \quad \text{(S13)}$$

**References:**


[21] Nardinocchi, P., Puntel, E. Finite bending solutions for layered gel beams. Int. J. Solids Struct., **2016**, 90, 228-235.
